# Genomics and Mapping of Teleostei (Bony Fish)

**DOI:** 10.1002/cfg.259

**Published:** 2003-04

**Authors:** Melody S. Clark

**Affiliations:** HGMP Resource Centre Genome Campus Hinxton, Cambridge CB2 4PP UK

## Abstract

Until recently, the Human Genome Project held centre stage in the press releases concerning sequencing programmes. However, in October 2001, it was announced that the Japanese puffer fish (*Takifugu rubripes, Fugu*) was the second vertebrate organism to be sequenced to draft quality. Briefly, the spotlight was on fish genomes. There are currently two other fish species undergoing intensive sequencing, the green spotted puffer fish (*Tetraodon nigroviridis*) and the zebrafish (*Danio rerio*). But this trio are, in many ways, atypical representations of the current state of fish genomic research. The aim of this brief review is to demonstrate the complexity of fish as a group of vertebrates and to publicize the ‘lesser-known’ species, all of which have something to offer.


**Comparative and Functional Genomics**
*Comp Funct Genom* 2003; **4**: 182–193.
Published online 1 April 2003 in Wiley InterScience (www.interscience.wiley.com). **DOI:** 10.1002/cfg.259

**Featured Organism**

### Genomics and mapping of Teleostei (bony fish)

Melody S. Clark*
*HGMP Resource Centre, Genome Campus, Hinxton, Cambridge CB2 4PP, UK*

**Correspondence to: Melody S. Clark, HGMP Resource Centre, Genome Campus, Hinxton, Cambridge CB2 4PP, UK. E-mail: mclark@hgmp.mrc.ac.uk*

Received: 10 November 2002
Revised: 5 December 2002
Accepted: 28 January 2003

### Abstract

**Until recently, the Human Genome Project held centre stage in the press releases concerning sequencing programmes. However, in October 2001, it was announced that the Japanese puffer fish (*Takifugu rubripes, Fugu*) was the second vertebrate organism to be sequenced to draft quality. Briefly, the spotlight was on fish genomes. There are currently two other fish species undergoing intensive sequencing, the green spotted puffer fish (*Tetraodon nigroviridis*) and the zebrafish (*Danio rerio*). But this trio are, in many ways, atypical representations of the current state of fish genomic research. The aim of this brief review is to demonstrate the complexity of fish as a group of vertebrates and to publicize the 'lesser-known' species, all of which have something to offer. Copyright © 2003 John Wiley & Sons, Ltd.**

**Keywords:** **Teleostei; fish; genomics; BACs; sequencing; aquaculture**

### Background

Fish have the potential to be immensely useful model organisms in medical research, as evidenced by the genomic sequencing programmes mentioned above. Indeed, there is an increasing number of alternative species, such as *Xiphophorus* and medaka, which are being promoted in this area. However, it is fair to say that, in general, fish are the poor relations of high-throughput molecular biology. To put fish into context, they comprise over half of all known vertebrates and are economically very important. They are a significant source of revenue, with the fisheries industry (national fishing fleets, aquaculture and associated processing) generating €20 billion per year for the EU alone (without taking into account recreational or game fishing and aquarium supplies). This contrasts with the fact that the species undergoing sequencing programmes were chosen due to their potential as model genomes/organisms, rather than their commercial importance. Globally in aquaculture, the three most important fish are carp, Atlantic salmon and trout; with anchoveta, Alaska pollock and Chilean jack mackerel leading the wild-caught fisheries production figures. The equivalents within the EU are trout, Atlantic salmon and sea bass/bream for aquaculture; with herring, mackerel and sprat for wild-caught fisheries. None of these species is the subject of a high-profile genomics programme.

### Fish relationships

The term 'fish' is not a taxonomic rank, but a convenient label for a diverse group of organisms (for a comprehensive review, see Nelson, 1994). Overall, this convenient grouping of 'fish' varies depending on different sources, but can include the jawless vertebrates (Agnatha), sharks and rays (Chondrichthyes), the lobe-finned fishes (Sarcopterygii) and the ray-finned fishes (Actinopterygii, including the Teleostei).

The lobe-finned fishes are in an evolutionarily critical position leading to the human lineage. The ray-finned fishes diverged from this 'main' lineage approximately 450 million years ago and have since undergone massive diversification in morphology, physiology and habitat. Their

Copyright © 2003 John Wiley & Sons, Ltd.

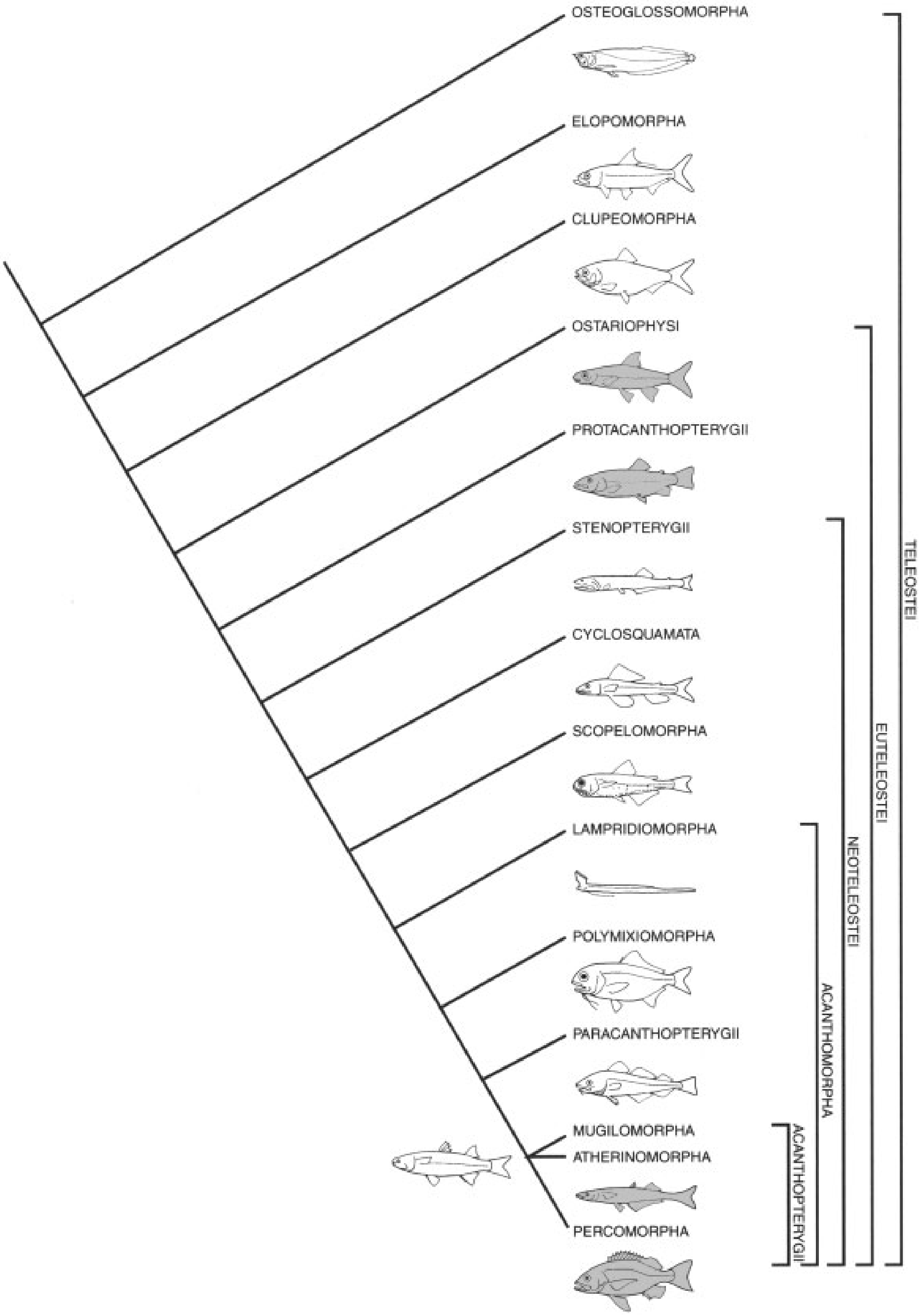

**Figure 1.** Phylogenetic relationships among living teleosts. Fish coloured in grey represent orders in which species are undergoing intensive mapping or sequencing programmes. Reproduced from *The Diversity of Fishes* by Helfman, Collette and Facey, by permission of Blackwell Science

Copyright © 2003 John Wiley & Sons, Ltd. 

genomes did not remain static and they are still evolving, with the phylogenetic relationships uncertain in many cases. Within this particular class (Actinopterygii) are those regarded as the more 'ancient fishes'. This latter category includes the sturgeons, paddlefishes and bichirs, which have relatively few extant members when compared to the rest of the class. This review will be largely restricted to a sub-set of the ray-finned fishes, the Teleostei or bony fishes (Figure 1), where the model and (most) commercial species are found. Table 1 lists the more commonly known members of each order (Nelson, 1994).

### Genome sizes and karyotypes

Fish certainly appear to have a much more dynamic and plastic genome than that of mammals, with genome sizes varying from 400 Mb in some of the Tetraodontidae to over 1000 Mb in the African lungfishes (Hinegardner, 1968; Hinegardner and Rosen, 1972; Ohno, 1974; Tiersch *et al.*, 1989). This wide range of genome sizes is also reflected in huge karyotypic variation, with diploid numbers as low as $2n = 22-26$ in some Nototheriidae (Ozouf-Costaz *et al.*, 1997), up to $2n = 240-260$ in some anadromous Acipenseridae (Fontana *et al.*, 1997). However, these diploid numbers hide the fact that many species are polyploid. Although the salmonids are the best known example of such, many other species, such as members of the Cobitidae, Catostomidae and Asipenseridae also contain different ploidy levels, even up to $8\times$ (Ohno, 1974; Bailey *et al.*, 1978, and references therein). Hence fish, as a group of vertebrates, do not seem to have the same stringent genomic controls that exist within other groups of vertebrates, a property which may be due in part to their lack of a rigid sex chromosome system. Data on the more common species is given in Table 2.

### Polyploidy and fish-specific duplications

It is known that many fish are polyploid, the prime example given above being that of the salmonids, in which members such as trout and salmon are actually partial tetraploids, $2n = 4\times$ (Lee and Wright, 1981; Wright *et al.*, 1983; Allendorf and Thorgaard, 1984). The term 'partial' means that the species have undergone an ancient extra whole genome duplication (i.e. in addition to the two rounds of whole genome duplication which occurred in the vertebrate lineage, proposed by Ohno, 1970) and are currently reverting to diploidy via a process of gene loss. However, there is currently much debate as to whether the whole of the Euteleostei have undergone an extra whole genome duplication, or just isolated

**Table 1.** Main orders of the Teleostei accompanied by their more commonly known family members. Most examples are listed against the Percomorpha, as these comprise approximately 50% of all known extant fishes. Species discussed in this review are also indicated

| Teleostei sub-division | Common families | Family members |
|---|---|---|
| Osteoglossomorpha | Elephantfishes, mooneyes | |
| Elopomorpha | Tarpons, eels | |
| Clupeomorpha | Herrings, anchovies | |
| Ostariophysi | Carps, suckers, loaches, catfishes | Catfish, zebrafish |
| Protocanthopterygii | Pikes, mudminnows, smelts, salmonids | Salmon, trout |
| Stenopterygii | Lightfishes, bristlemouths | |
| Cyclosquamata | Telescopefishes, greeneyes | |
| Scopelomorpha | Lanternfishes | |
| Lampridiomorpha | Ribbonfishes, oarfishes | |
| Polymixiomorpha | Beardfishes | |
| Paracanthopterygii | Cavefishes, cods, hakes, rattails, anglerfishes | |
| Mugilomorpha | Mullets | |
| Atherinomorpha | Killifishes, rainbowfishes, flying fishes, silversides, medakas | Medaka, *Xiphophorus* |
| Percomorpha | Basses, perches, jacks, chubs, snappers, cichlids, mackerels, flounders, pufferfishes, swordfishes, tunas | Tilapia, *Tetraodon, Fugu,* sea bass, sea bream |

Copyright © 2003 John Wiley & Sons, Ltd. 

**Table 2.** Fish genome sizes and chromosome complements of some of the more commercially important species. X indicates data not known. Where possible, species discussed in this review have been included. Brackets indicate that the chromosome number given is that of a closely related family member, not the exact species named (data taken from Bejar *et al.*, 1997; Hinegardner, 1968; Hinegardner and Rosen, 1972; Ohno, 1974; Sola *et al.*, 1993; Tiersch *et al.*, 1989; **http://www.genomesize.com/fish.htm; http://www.fishbase.org**; Angelo Libertini, personal communication). In perspective; the coho salmon, carp and pacific herring are 90%, 50% and 28%, respectively, of the size of the human genome

| Sub-division | Latin name | Common name | Haploid DNA content (Mb) | Diploid chromosome no. |
|---|---|---|---|---|
| *Elopomorpha* | *Anguilla rostrata* | Atlantic eel | 1400 | 38 |
| Clupeomorpha | *Engraulis mordax* | Californian anchovy | 1500 | 46–48 |
| | *Clupea pallasi* | Pacific herring | 770 | 52 |
| Ostariophysi | *Cyprinus carpio* | Common carp | 1700 | 50 |
| | *Danio rerio* | Zebrafish | 1800 | 50 |
| | *Ictarulus punctatus* | Channel catfish | 1050 | 58 |
| Protocanthopterygii | *Onchorhynchus kisutch* | Coho salmon | 3000 | 60 |
| | *Onchorhynchus tshawytscha* | Chinook salmon | 3300 | 68 |
| | *Salmo salar* | Atlantic salmon | 3100 | 60 |
| | *Onchorhynchus mykiss* | Rainbow trout | 2600 | 60 |
| | *Salmo trutta* | Brown trout | 2800 | 80 |
| Paracanthopterygii | *Merluccinus bilinearis* | Silver hake | 930 | — |
| Atherinomorpha | *Oryzias latipes* | Medaka | 800 | 48 |
| | *Xiphophorus maculatus* | *Xiphophorus* | 950 | 48 |
| Percomorpha | *Dicentrarchus labrax* | European sea bass | 1600 | 48 |
| | *Lutjanus campechanus* | Red snapper | 1400 | — |
| | *Sparus aurata* | Gilthead sea bream | 1650 | 48 |
| | *Tilapia nilotica* | Nile tilapia | 1200 | 44 |
| | *Scomber scombrus* | Atlantic mackerel | 970 | (48) |
| | *Pseudopleuonectes americus* | Winter flounder | 700 | 48 |
| | *Fugu rubripes* | Japanese pufferfish | 365 | 44 |
| | *Tetraodon nigroviridis* | Freshwater Pufferfish | 380 | 42 |

species. This debate arose mainly from the results of mapping studies of zebrafish, which showed that approximately 25% of loci are duplicated (Gates *et al.*, 1999 and references therein; Barbazuk *et al.*, 2000) and led to the proposal that this is also a partial tetraploid (Amores *et al.*, 1998; Woods *et al.*, 2000; Postlethwait *et al.*, 2000; Babuzuk *et al.*, 2000). Indeed, as molecular studies on fish expand, many 'extra' genes are being discovered in this class of vertebrates (Wittbrodt *et al.*, 1998). The exact origin of these 'extra' genes is hotly debated, with two main camps; those that believe that these genes arose due to a basal (extra) whole genome duplication in the Euteleostei (Taylor *et al.*, 2001a,b) and those who take the view that many different independent gene or chromosomal duplications have occurred in the fish lineage (Robinson-Rechavi *et al.*, 2001a,b,c; Hughes *et al.*, 2001). It is doubtful whether there will ever be complete agreement between the two sides, even with the imminent sequencing of three fish genomes.

### The choice of current fish sequencing models

So why is it that the second draft vertebrate genome is that of an infamous, potentially deadly fish, available only in Japan? The answer is largely historical. When *Fugu* was originally proposed as a model genome, over 10 years ago, the high-throughput sequencing technologies were just being developed to cope with the sequencing of yeast and *C. elegans*. The complete sequence of the human genome was viewed as a very distant possibility and work concentrated on EST programmes and sequencing

Copyright © 2003 John Wiley & Sons, Ltd.

of individual genes. *Fugu* was proposed as a 'cut-price' vertebrate, with a genome one-eighth the size of human but with a similar repertoire of genes and a potential bridge between the sequence of the nematode and human. However, in a bizarre twist of events, the human genome was completed first and, with a requirement to fuel the enormous world-wide sequencing capacity, *Fugu* was retrospectively sequenced.

*Tetraodon* is a freshwater species and therefore more easily maintained when compared with the marine *Fugu*. More importantly, it is readily available in most aquarium shops and this was proposed as a more accessible source of material, hence prompting the sequencing programme at Genoscope. Additionally, *Tetraodon* can be kept in a tank on the laboratory windowsill, whereas *Fugu* is only available from Japan, and grows up to 1 kg in the first year, necessitating swimming pool-sized aquaria as a minimum. However, both *Fugu* and *Tetraodon* will only be used as model genomes, as neither breeds easily (if at all) in captivity, therefore ruling out linkage maps, transgenics, inbred lines, etc. They will mainly be used as models for *in silico* comparisons with human, aiding gene prediction (Roest Crollius *et al*., 2000) and identification of conserved non-coding regulatory motifs (Rothenberg, 2001). It is intended to complete the *Fugu* genome to reference standard; however, the whole genome shotgun of *Tetraodon* currently stands at 8.3× coverage (H. Roest Crollius, personal communication) with, as yet, no publicized aim of finishing the complete genome.

Zebrafish differs from the previous two fish in that it breeds easily and is very amenable to manipulation. It is used as a developmental model, due to the transparent nature of the embryos (Nusslein-Volhard, 1994) and is very popular in medical comparative functional genomics studies (Dodd *et al*., 2000; Briggs, 2002). As an organism, it is very amenable to ENU mutagenesis technology, with two large screening programmes producing numerous mutants of medical importance (Solnica-Krezel *et al*., 1994; Driever *et al*., 1996; Haffter *et al*., 1996). Whilst the genome is one-third the size of human, it is still intended to sequence this organism to reference standard within the next year or two. Whilst these three fish are not necessarily representative species of the whole grouping, they have raised the profile of fish as models within medical research.

### Contribution of other fish species

Fish are an immensely diverse group of organisms, inhabiting an enormous variety of habitats. Some live in almost pure freshwater, whilst others survive in very salty lakes at three times the salinity of seawater. Certain tilapia species can live quite happily in hot soda lakes with a temperature of 44 °C; conversely, the cod icefishes prefer around −2 °C (Nelson, 1994). The consequential protein diversity is potentially fascinating, both from an evolutionary point of view and also for pharmaceutical exploitation. The classic example of the latter, so far, is the increased potency of salmon calcitonin and its use as a therapeutic agent for inhibiting calcium loss from bone in humans (Wisneski, 1990).

At the moment, fish are contributing significantly to medical research. They provide a wide range of experimental tumour models, which are cheaper and easier to keep than mammalian models. Also they can be bred in such numbers to produce statistically meaningful results. It has long been known that *Xiphophorus* interspecies hybrids provide genetically controlled models of cancer formation [extensively reviewed in a special edition of *Marine Biotechnology* 2001; **3**(1)]. However, a survey of the archives of the Registry of Tumours in Lower Animals (RTLA; Harshbarger and Slatick, 2001) uncovered a list of over 215 cultured fish species, which display a broad range of spontaneous or induced tumours. These represent a valuable collection for finding appropriate surrogates for research with which to enhance our knowledge of carcinogenesis and other human diseases. Some of the lesser-known models include carcinoma of the urinary bladder in oscar (*Astronotus ocellatus*; Petervary *et al*., 1996) and nephroblastoma resembling Wilm's tumour in Japanese eels (*Anguilla japonica*; Masahito *et al*., 1992). Medaka is also being using in tumour genetics (Rotchell *et al*., 2001), but has been promoted more specifically for the study of germ cell mutagenesis and genomic instability (Shima and Shimada, 2001). One example of the latter is its use to estimate germ cell mutations in Astronauts exposed to high atomic number, high-energy (HZE) nuclei present in cosmic rays (Setlow and Woodhead, 2001). In addition to these specific medical uses, fish are also valuable tools for deciphering essential biological processes (Bolis *et al*., 2001; Clark *et al*., 2002; Grunwald and Eisen, 2002; Korpi *et al*., 2002).

Copyright © 2003 John Wiley & Sons, Ltd.

Fish have many advantages over mammals for research purposes. Many are small, with short reproductive cycles and are relatively easy to maintain. Therefore they are ideally suited to lifetime, multigenerational and population studies. One particular high-profile use is that of endocrine disrupters and environmental monitoring of toxic compounds (often called 'biomonitoring'; Bailey 1996, 2000; Bonaventura, 1999). The variety within fish species allows the researcher to pinpoint the most susceptible species for each particular compound under study. Rainbow trout have a long history of such a use (extensively reviewed in Thorgaard *et al*., 2002) and an increasing number of different fish are being employed to monitor our increasingly polluted environment. These include the three-spined stickle back, sheepshead minnow, sunshine bass and medaka to measure environmental oestrogens (Katsiadaki *et al*., 2002; Larkin *et al*., 2002; Todorov *et al*., 2002; Metcalfe *et al*., 2001). A number of other fish species can be used to monitor chemicals such as dioxins, polycyclic aromatic hydrocarbons (PAHs), polyhalogenated biphenyls (PCBs), alkylphenols, DDT isomers, etc. (Bailey *et al*., 1996; Ballatori and Villalobos, 2002; Fent, 2001; Hahn, 2001; Thorgaard *et al*., 2002; Wester *et al*., 2002).

Medical and environmental models aside, it cannot be denied that the main commercial purpose of fish is as a source of food. However, the world's fisheries are in crisis, with serious discussion within Europe of a total ban on capturing certain species, such as cod and haddock, to prevent their extinction. This comes at a time when there is growing concern over the health of the European population, with problems such as clinical obesity becoming more common and fish are being promoted as part of a 'healthier' diet. The nutritional benefits of a balanced diet, which includes seafood, are well known. Seafood contains a range of ingredients that have a positive effect on health and which, through the premise of 'functional' food, could be enhanced to meet a response for these needs. There is an increasing world deficit between supply and demand for fish and fish products, which is only partially being met through aquaculture. Atlantic salmon and trout are world leaders in fish aquaculture, with catfish the major aquaculture species in the USA and the two Spiridae, sea bream and sea bass, particular European favourites. Whilst aquaculture systems have long been established for some species, there are still considerable problems concerning environmental pollution, diet quality and rearing difficulties, involving a high incidence of skeletal abnormalities and captive stress. Biotechnology, including genomics programmes, can aid in our understanding of these problems and optimization of production processes. Although mapping programmes exist for the most important aquaculture species, our genetic knowledge of wild-caught fisheries stock is comparatively low. The latter is particularly disturbing, as many species are either at, or approaching, their minimum levels of sustainability in the wild and there are few markers with which to monitor population structure and associated genetic bottlenecks. It also means that, should captive breeding programmes be initiated, there are not sufficient tools to rapidly develop marker-assisted selection breeding programmes.

### Tools for fish mapping and genome sequencing

### ESTs

A relatively quick and easy way to generate gene data from any species is via the construction and sequencing of EST libraries. Indeed, this has been carried out for many fish species including winter flounder (Douglas *et al*., 1999), tilapia (Hamilton *et al*., 2000), Japanese eel (Miyahara *et al*., 2000), catfish (Ju *et al*., 2000; Cao *et al*., 2001; Karsi *et al*., 2002) and salmon (Davey *et al*., 2001), although many ESTs find their way into the public databases (GenBank and EMBL) without being written up for publication. All contribute to our knowledge on protein diversity in fish and provide markers for placement on genetic maps and annotation data for genomic sequence. ESTs also provide the raw clones for the development of microarrays, a potentially very powerful tool for expression analysis.

### BACs

Large insert libraries, such as BACs, are essential tools in any genome sequencing project. BAC libraries are increasingly being produced for fish species, including red seabream (Katagiri *et al*., 2002), rainbow trout, carp and tilapia (Katagiri *et al*., 2001) and medaka (Matsuda *et al*., 2001).

Copyright © 2003 John Wiley & Sons, Ltd. 

BACs can provide useful data on short-range linkage and a tool from which to genomically clone sequences of interest. The latter is of particular use for studying regulatory elements and control regions. A fingerprinted BAC library can provide the framework for a directed sequencing programme on any scale. Whilst whole genome shotguns (WGS) are effective for smaller fish genomes, the problem of producing contigs of useful size by this method becomes increasingly complex with the larger genomes, as the amount of 'junk' DNA increases and the problem of polyploidy has to be addressed. Most of the highly repeated elements have to be removed from the WGS dataset to prevent erroneous joining of fragments. Certainly it has been found to be advantageous to use only one animal, if possible, in the construction of the libraries, and indeed sequencing programmes, to minimize problems of polymorphic variation between individuals.

### Linkage maps

These provide valuable tools for the positional cloning of genes and analysis of complex traits (QTLs) and also act as a useful reference framework for genome sequencing studies. However, they do require the production of inbred lines and the development of a set of polymorphic markers. In fish, a wide range of marker types has been used: amplified fragment length polymorphisms (AFLPs), randomly amplified polymorphic DNA (RAPD); intervening repeat sequences (IRSs); expressed sequence tags (ESTs); sequence tagged sites (STSs); interspersed nuclear repeats (INRs); simple sequence repeats (SSRs); variable number tandem repeats (VNTRs); short interspersed elements (SINEs) and expressed sequence marker polymorphisms (ESMPs). These have been very effective at promoting genetic analysis and building detailed maps for a number of species, such as zebrafish (Woods *et al.*, 2000; Barbazuk *et al.*, 2000), catfish (Liu *et al.*, 1999a,b,c), trout (Young *et al.*, 1998; Sakamoto *et al.*, 2000), medaka (Naruse *et al.*, 2000), tilapia (Kocher *et al.*, 1998, Agresti *et al.*, 2000; McConnell *et al.*, 2000), salmon (Linder *et al.*, 2000) and *Xiphophorus* (Kazianis *et al.*, 1996). The result is that transfer of markers between species and comparison of map data between species is difficult. Genomic sequencing and the development of gene markers will circumnavigate this problem.

The issue of 'extra' genes and different ploidy levels does represent a potential problem for the development of markers and a genetic map. In salmon, some of the markers are duplicated, i.e. they show up to four alleles and cause problems with genotyping. These effectively have to be ignored when scoring the genotypes and so the tetraploid areas are under-represented in the genetic map. BAC contigs and SNPs used in conjunction with genotyping may help resolve this (Hoyheim, personal communication). This is an issue that will arise with other fish species.

#### Microarray technology

Whilst a relatively recent technology, published uses include studying cold acclimation in catfish (Ju *et al.*, 2002) and the use of sheepshead minnows for environmental monitoring (Larkin *et al.*, 2002). There are microarrays currently being developed for most of the 'popular' fish species, such as Atlantic salmon (w. Davidson, personal communication.) and sea bream (M. S. Clark, unpublished). With the large number of fish ESTs available in the public databanks and numerous libraries distributed in labs world-wide, microarrays will inevitably be targeted in new projects and provide valuable insights into fish biochemistry and physiology.

Linkage maps are usually the first tool to be developed in fish genomics, due to the relative ease of manipulating the fish and producing inbred and backcross lines. These are also comparatively cheaper than launching straight into a genome sequencing programme. However, the spin-off from the human genome project is that genomics techniques, such as the development of BAC libraries, are more readily available and cheaper than ever before. A genome programme (although impressive on the grant proposals) is not always the best option in many cases. QTL mapping of commercially important traits may be more efficiently achieved using crosses between contrasting fish populations, or expression analysis using microarray technology.

### Progress in mapping and sequencing fish genomes

The sequence information on many fish is sporadic, often restricted to a few particular genes or

Copyright © 2003 John Wiley & Sons, Ltd.

microsatellites. However, it is possible to identify the front-runners in genomics studies, which represent the best candidates for genomic sequencing in the near future. Websites have been given for some of the BAC libraries in the various species. However, please note that these may not necessarily represent the particular libraries being used in the mapping projects described.

### Atlantic salmon

*Linkage map*: 522 microsatellite markers representing 28 linkage groups (plus two small ones consisting of two markers each). There are 29 linkage groups expected in the European strain.
*BAC library*: constructed.
*BAC library available*: **http://www.chori.org/bacpac/salmon214.htm**
*BAC contig map*: Fingerprint map representing 15× coverage under construction at BC Cancer Agency's Genome Sciences Centre, Canada.
*Genome sequencing*: none planned at present.

### Catfish

*Linkage map*: 454 markers, resolved into 43 linkage groups ($2n = 2\times = 58$).
*BAC library*: constructed.
*BAC library available*: **http://www.chori.org/bacpac/catfish212.htm**
*BAC contig map*: none planned at present.
*Genome sequencing*: none planned at present.

### Medaka

*Linkage map*: 1300 markers have been mapped, representing an average marker distance of less than 0.85 cM.
*BAC libraries*: Constructed from the Southern and Northern strains of medaka.
*BAC library available*: **http://www.rzpd.de** and **http://biol1.bio.nagoya-u.ac.jp:8000/bac-lib.html**
*BAC contig map*: currently under progress at the Max-Planck Institute.
*Genome sequencing*: commenced August 2002, 1× WGS (whole genome shotgun) due end of 2002, 5× WGS due to start early 2003. Sequencing Centre, National Institute of Genetics, Japan.

### Sea bass/sea bream

*Linkage maps*: development of microsatellite and EST markers in progress.
*BAC libraries*: Developed for EU Consortium use.
*BAC contig map*: none available.
*Genome sequencing*: none planned at present.

### Tilapia

*Linkage map*: 550 microsatellites and 15 genes.
*BAC library*: constructed.
*BAC library available*: **http://hcgs.unh.edu/BAC/**
*BAC contig map*: 22 000 already fingerprinted with plans to complete 35 000 clones (5× genome coverage by the end of 2002).
*Genome sequencing*: none planned at present.

### Trout

*Linkage map*: two maps have been produced; that of Young *et al.* (1998) comprises 476 markers segregated into 31 major linkage groups and 11 small groups and Sakamoto *et al.* (2000) has 109 markers segregating into 29 linkage groups. $2n = 2\times = 60$.
*BAC library*: four have been constructed, two in Japan (Katagiri *et al.*, 2001), coverage 5.3× and 6.7×, and two in the USA, coverage 4× and 10×.
*BAC library available*: **http://www.genomex.com/AEX_zone/AEX_BAC_Library_List.xls**
*BAC contig map*: none planned at present.
*Genome sequencing*: none planned at present.

### *Xiphophorus*

*Linkage map*: two recombination-based maps have been produced in hybrid backcross lines. The first was constructed using a cross between *X. maculatus* and *X. helleri*; it comprises 320 markers (mainly RAPDs with some isozyme and microsatellites), which provides approximately 8.2 cM coverage and segregates in 24 linkage groups. The second was created using a cross between *X. maculatus* and *X. andersi* and comprises approximately 220 microsatellite loci, 38 isozyme loci and a limited number of cloned genes. This map is still being worked on (Kazianis, personal communication).
*BAC library*: constructed.
*BAC contig map*: not available.
*Genome sequencing*: none planned at present.

### Prospects

The phylogenetic juxtaposition of the three species currently undergoing sequencing may prove pivotal to the expansion of fish genomics research.

Copyright © 2003 John Wiley & Sons, Ltd.

Zebrafish is relatively distant (Ostariophysi) from the two pufferfish species (Percomorpha) and it will be interesting to evaluate how similar gene structure and gene positioning are within the same order (Percomorpha) and within different euteleost orders (Ostariophysi vs. Percomorpha; Figure 1). This should provide a reasonable gauge of evolutionary change within fish and therefore the potential for data mining of model species with regard to other fish. If gene structures and orders are significantly different between zebrafish and the pufferfish, this will add to the pressure to sequence (at least to draft quality) additional species. At a minimum, this should include a member of the salmonids for their commercial importance, a marine perciform, again for their commercial importance but also because most marine species have a chromosome complement of $2n = 48$, unlike the model species now under study, and a strong case could be made for one of the fish cancer models. Fish have a lot to offer to humans, not only in terms of health (diet and medication) but also in terms of our guardianship of the environment — sequencing a range of fish genomes would certainly help to unlock that potential. One thing is certain: fish genomics now has a higher profile and a greater number of tools than ever before.

### Acknowledgements

A big thank you to the following people for their help and provision of data: Luca Bargelloni, Hughes Roest Crollius, Willie Davidson, Hiroshi Hori, Bjorn Hoyheim, Steve Kazianis, Tom Kocher, Angelo Libertini, John Liu, Gary Thorgaard, Filip Volckaert, Ron Walter; also to Jo Wixon for critical reading of the manuscript.

### Web-based resources

### General Information on fish, sequencing projects and phylogeny

#### Fishbase

Main site: **www.fishbase.org**
French mirror site: **http://ichtyonbl.mnhn.fr/**
German mirror site: **http://filaman.uni-kiel.de/**
The global information system with everything you ever wanted to know about fishes. Contains an excellent search facility (by common or Latin name) and lists numerous facts for each fish, such as importance, distribution, environment, genetics, etc.

#### Larvalbase

**http//www.larvalbase.org**: developed in close conjunction with Fishbase and contains comprehensive information on fish larvae which are relevant in the field of fisheries research and finfish culture. Is a similar format to Fishbase.

#### FAO fisheries web site

**http://www.fao.org/fi/default.asp**
Major site containing world production figures and statistics, plus numerous reports on the state and management of world fisheries.

#### Tree of Life

**http://tolweb.org/phylogeny.html**
Work out the phylogenetic relationships of your favourite fish with this web site.

#### Animal genome size database

**http://www.genomesize.com/fish.htm**
Find out the genome size and chromosome number of your favourite fish.

#### GOLD™ Genomes OnLine Database

**http://igweb.integratedgenomics.com/GOLD/#1**
This site lists all complete and ongoing genome projects.

### Genome mapping and sequencing information on specific fish species

#### Catfish

**http://www.ag.auburn.edu/dept/faa/index.htm**
The home page of Department of Fisheries and Allied Aquacultures at Auburn University. Access to recent publications and staff listings, plus brief research resumés.

#### Fugu

ENSEMBL: **www.ensembl.org/Fugu_rubripes**
HGMP, UK: **http://www.fugu.hgmp.mrc.ac.uk**
Institute of Molecular and Cellular Biology, Singapore: **http://www.fugu-sg.org**

Copyright © 2003 John Wiley & Sons, Ltd. 

Joint Genome Institute, USA: **http://genome.jgi-psf.org/fugu6/fugu6.home.html**
The *Fugu* draft sequence information is available on four sites at the moment, all with different sequence viewers, so take your pick.

#### Medaka

**http://bio11.bio.nagoya-u.ac.jp:8000/**
Home of the medaka genome project with many links to research projects and resources.

#### Salmonids

**http://locus.jouy.inra.fr/cgi-bin/lgbc/mapping/common/intro2.pl?BASE=rainbow**
INRA Rainmap database for the mapping of the rainbow trout genome.
**http://www.thearkdb.org/**
Salmon mapping database based at the Roslin Institute, UK.
**http://www.bcgsc.bc.ca/gc/salmon.shtml**
The Genome Sciences Centre, Canada: Genomics of Atlantic salmon home page.

#### Sea bass

**www.bassmap.org**
This is the official site of the EU sponsored project, listing aims, participants and downloadable reports.

#### Sea bream

**www.intelligence.tuc.gr/~bridgemap**
Another EU-funded project. The site provides information on project objectives, participants and achievements.

#### Tetraodon

Genoscope, France: **www.genoscope.cns.fr/externe/English/Projets/Projet_C/C.html**
Whitehead Institute, USA: **www-genome.wi.mit.edu/annotation/tetraodon**
The *Tetraodon* draft sequence data is available on two sites, with an option on the French language version at Genoscope.

#### Tilapia

**http://tilapia.unh.edu/WWWPages/TGP/TGP.html**
This site describes current research projects and map status, plus resources available and links to tilapia aquaculture and recipes.
**http://www.thearkdb.org/**
Tilapia mapping database based at the Roslin Institute, UK.

#### Xiphophorus

**www.xiphophorus.org**
Home page of the *Xiphophorus* Genetic Stock based at Southwest Texas State University. Contains details of current research programmes, contact details for live fish requests and related sites including several for hobbyists.

#### Zebrafish

ZFIN: **http://zfin.org/cgi-bin/webdriver?Mival=aa-ZDB_home.apg**
ENSEMBL: **www.ensembl.org/Danio_rerio**
Sanger Institute, UK: **www.sanger.ac.uk/Projects/D_rerio/**
ZFIN is an extensive database of information for zebrafish researchers which aims to integrate zebrafish genetic, genomic and developmental information. ENSEMBL contains the latest draft sequence of the zebrafish genome, whilst the Sanger site provides a more comprehensive service with latest news, data downloads, mapping status, resources available and descriptions of teams and people.

### References

Agresti JJ, Seki S, Cnaani A, *et al.* 2000. Breeding new strains of tilapia: development of an artificial center of origin and linkage map based on AFLP and microsatellite loci. *Aquaculture* **185**: 43–56.

Allendorf FW, Thorgaard GH. 1984. Tetraploidy and the evolution of salmonid fishes. In *Evolutionary Genetics of Fishes*, Turner BJ (ed.). Plenum: New York; 1–46.

Amores A, Force A, Yan YL, *et al.* 1998. Zebrafish hox clusters and vertebrate genome evolution. *Science* **282**: 1711–1714.

Bailey GS, Poulter RTM, Stockwell PA. 1978. Gene duplication in tetraploid fish: model for gene silencing at unlinked duplicate loci. *Proc Natl Acad Sci USA* **75**: 5575–5579.

Bailey GS, Williams DE, Hendricks JD. 1996. Fish models for environmental carcinogenesis: the rainbow trout. *Environ Health Perspect* **104**: 5–21.

Ballatori N, Villalobos AR. 2002. Defining the molecular and cellular basis of toxicity using comparative models. *Toxicol Appl Pharmacol* **183**: 207–220.

Copyright © 2003 John Wiley & Sons, Ltd.

Barbazuk WB, Korf I, Kadavi C, *et al*. 2000. The syntenic relationship of the zebrafish and human genomes. *Genome Res* **10**: 1351–1358.

Bejar J, Borrego JJ, Alvarez MC. 1997. A continuous cell line from the cultured marine fish gilt-head sea bream (*Sparus aurata* L.). *Aquaculture* **150**: 143–153.

Bolis CL, Piccolella M, Dalla Valle AZ, Rankin JC. 2001. Fish as models in pharmacological and biological research. *Pharmacol Res* **44**: 265–280.

Bonaventura C. 1999. NIEHS workshop: unique marine/freshwater models for environmental health research. *Environ Health Perspect* **107**: 89–92.

Briggs JP. 2002. The zebrafish: a new model organism for integrative physiology. *Am J Physiol* **282**: R3–R9.

Cao D, Kocabas A, Ju Z, *et al*. 2001. Transcriptome of channel catfish (*Ictalurus punctatus*): Initial analysis of genes and expression profiles of the head kidney. *Animal Genet* **32**: 169–188.

Clark MS, Ingleton PM, Power DM. 2002. The application of comparative genomics to fish endocrinology. *Int Rev Cytol* (in press).

Davey GC, Caplice NC, Martin SA, Powel R. 2001. A survey of genes in the Atlantic salmon (*Salmo salar*) as identified by expressed sequence tags. *Gene* **263**: 121–130.

Dodd A, Curtis PM, Williams LC, Love DR. 2000. Zebrafish: bridging the gap between development and disease. *Hum Mol Genet* **9**: 2443–2449.

Douglas SE, Gallant JW, Bullerwell CE, *et al*. 1999. Winter flounder expressed sequence tags: establishment of an EST database and identification of novel fish genes. *Mar Biotechnol* **1**: 458–464.

Driever W, Solnica-Krezel L, Schier AF, *et al*. 1996. A genetic screen for mutations affecting embryogenesis in zebrafish. *Development* **123**: 37–46.

Fent K. 2001. Fish cell lines as versatile tools in ecotoxicology: assessment of cytotoxicology, cytochrome P4501A induction potential and estrogenic activity of chemicals and environmental samples. *Toxicol In Vitro* **15**: 477–488.

Fontana F, Rossi R, Lanfredi M, Arlati G, Bronzi P. 1997. Cytogenetic characterization of cell lines from three sturgeon species. *Caryologia* **50**: 91–95.

Gates MA, Kim L, Egan ES, *et al*. 1999. A genetic linkage map for zebrafish, comparative analysis and localization of genes and expressed sequences. *Genome Res* **9**: 334–347.

Grunwald DJ, Eisen JS. 2002. Timeline — headwaters of the zebrafish: emergence of a new model vertebrate. *Nature Rev Genet* **3**: 717–724.

Haffter P, Granato M, Brand M, *et al*. 1996. The identification of genes with unique and essential functions in the development of the zebrafish, *Danio rerio*. *Development* **123**: 1–36.

Hahn ME. 2001. Dioxin toxicology and the aryl hydrocarbon receptor: insights from fish and other non-traditional models. *Mar Biotechnol* **3**: S224–S238.

Hamilton LC, MacPherson GR, Wright JM. 2000. Exposed sequence tags derived from brain tissue of *Oreochromis niloticus*. *J Fish Biol* **56**: 219–222.

Harshbarger JH, Slatick MS. 2001. Lesser known aquarium fish tumour models. *Mar Biotechnol* **3**: S115–S129.

Hinegardner R. 1968. Evolution of cellular DNA content in teleost fishes. *Am Nature* **102**: 517–523.

Hinegardner R. 1976. The cellular DNA content of sharks, rays and some other fishes. *Comp Biochem Physiol B* **55**: 367–370.

Hinegardner R, Rosen DE. 1972. Cellular DNA content and the evolution of teleostean fishes. *Am Nature* **106**: 621–644.

Hughes AL, da Silva J, Friedman R. 2001. Ancient genome duplications did not structure the human hox-bearing chromosomes. *Genome Res* **11**: 771–780.

Ju Z, Dunham RA, Liu Z. 2002. Differential expression in the brain of channel catfish (*Ictalurus punctatus*) in response to cold acclimation. *Mol Genet Genom* **268**: 87–95.

Ju Z, Karsi A, Kocabas A, *et al*. 2000. Transcriptome analysis of channel catfish (*Ictalurus punctatus*): genes and expression profile from the brain. *Gene* **261**: 373–382.

Karsi A, Cao D, Li P, *et al*. 2002. Transcriptome analysis of channel catfish (*Ictalurus punctatus*): initial analysis of gene expression and microsatellite-containing cDNAs in the skin. *Gene* **285**: 157–168.

Katagiri T, Asakawa S, Minagawa S, *et al*. 2001. Construction and characterization of BAC libraries for three fish species; rainbow trout, carp and tilapia. *Anim Genet* **32**: 200–204.

Katagiri T, Minagawa S, Hirono I, *et al*. 2002. Construction of a BAC library for the red sea bream *Pagrus major*. *Fish Sci* **68**: 942–944.

Katsiadaki I, Scott AP, Mayer I. 2002. The potential of the three-spined stickleback (*Gasterosteus aculeatus* L.) as a combined biomarker for oestrogens and androgens in European waters. *Mar Environ Res* **54**: 725–728.

Kazianis S, Morizot DC, McEntire BB, Nairn RS, Borowsky RL. 1996. Genetic mapping in *Xiphophorus* hybrid fish, assignment of 43 AP-PCR/RAPD and isozyme markers to multipoint linkage groups. *Genome Res* **6**: 280–289.

Kocher TD, Lee WJ, Sobolewska H, Penman D, McAndrew B. 1998. A genetic linkage map of a cichlid fish, the tilapia, *Oreochromis niloticus*. *Genetics* **148**: 1225–1232.

Korpi ER, Grunder G, Luddens H. 2002. Drug interactions at GABA (A) receptors. *Prog Neurobiol* **67**: 113–159.

Larkin P, Folmar LC, Hemmer MJ, Poston AJ, Lee HS, Denslow ND. 2002. Array technology as a tool to monitor exposure of fish to xeno-estrogens. *Mar Environ Res* **54**: 395–399.

Lee GM, Wright JE Jr. 1981. Mitotic and meiotic analyses of brook trout, *Salvelinus fontinalis*. *J Hered* **72**: 321–327.

Linder KR, Seeb JE, Habicht C, *et al*. 2000. Gene-centromere mapping of 312 loci in pink salmon by half-tetrad analysis. *Genome* **43**: 538–549.

Liu Z, Karsi A, Dunham R. 1999a. Development of polymorphic EST markers suitable for genetic linkage mapping of catfish. *Mar Biotechnol* **1**: 437–447.

Liu Z, Li P, Argue BJ, Dunham RA. 1999b. Random amplified polymorphic DNA markers: usefulness for gene mapping and analysis of genetic variation of catfish. *Aquaculture* **174**: 59–68.

Liu Z, Li P, Kucuktas H, *et al*. 1999c. Development of amplified fragment length polymorphism (AFLP) markers suitable for genetic linkage mapping of catfish. *Trans Am Fish Soc* **128**: 317–327.

Masahito P, Ishikawa T, Okamoto N, Sugano H. 1992. Nephroblastomas in the Japanese eel *Anguilla japonica* Temminck and Schlegel. *Cancer Res* **52**: 2575–2579.

Copyright © 2003 John Wiley & Sons, Ltd. 

Matsuda M, Kawato N, Asakawa S, *et al*. 2001. Construction of a BAC library derived from the inbred Hd-rR strain of the teleost fish, *Oryzias latipes*. *Genes Genet Syst* **76**: 61–63.

McConnell SK, Beynon C, Leamon J, Skibinski DO. 2000. Microsatellite marker-based genetic linkage maps of *Oreochromis aureus* and *O. niloticus* (Cichlidae); extensive linkage group segment homologies revealed. *Anim Genet* **31**: 214–218.

Metcalfe CD, Metcalfe TL, Kiparissis Y, *et al*. 2001. Estrogenic potency of chemicals detected in sewage treatment plant effluents as determined by *in vivo* assays with Japanese medaka (*Oryzias latipes*). *Environ Toxicol Chem* **20**: 297–308.

Miyahara T, Hirono I, Aoki T. 2000. Analysis of expressed sequence tags from a Japanese eel *Anguilla japonica* spleen cDNA library. *Fish Sci* **66**: 257–260.

Naruse K, Fukamachi S, Mitani H, *et al*. 2000. A detailed linkage map of medaka, *Oryzias latipes*, comparative genomics and genome evolution. *Genetics* **154**: 1773–1784.

Nelson JS. 1994. *Fishes of the World*. Wiley: New York.

Nusslein-Volhard C. 1994. Of flies and fish. *Science* **266**: 572–574.

Ohno S. 1970. *Evolution by Gene Duplication*. Springer-Verlag: Berlin, New York.

Ohno S. 1974. *Animal Cytogenetics, Vol 4: Chordata 1: Protochordata, Cyclostomata and Pisces*. Gebrüder Borntraeger: Berlin, Stuttgart.

Ozouf-Costaz C, Pisano E, Thaeron C, Hureau J-C. 1997. Antarctic fish chromosome banding: significance for evolutionary studies. *Cybium* **21**: 399–409.

Petervary N, Gillette DM, Lewbart GA, Harshbarger JC. 1996. A spontaneous neoplasm of the renal collecting ducts in an oscar, *Astronotus ocellatus* (Cuvier), with comments on similar cases in this species. *J Fish Dis* **19**: 279–281.

Postlethwait JH, Woods IG, Ngo-Hazelett P, *et al*. 2000. Zebrafish comparative genomics and the origins of vertebrate chromosomes. *Genome Res* **10**: 1890–1902.

Robinson-Rechavi M, Laudet V. 2001a. Evolutionary rates of duplicate genes in fish and mammals. *Mol Biol Evol* **18**: 681–683.

Robinson-Rechavi M, Marchand O, Escriva H, Laudet V. 2001b. An ancestral whole-genome duplication may not have been responsible for the abundance of duplicated fish genes. *Curr Biol* **11**: 458–459.

Robinson-Rechavi M, Marchand O, Escriva H, *et al*. 2001c. Euteleost fish genomes are characterized by expansion of gene families. *Genome Res* **11**: 781–788.

Roest Crollius H, Jaillon O, Bernot A, *et al*. 2000. Estimate of human gene number provided by genome-wide analysis using *Tetraodon nigroviridis* DNA sequence. *Nature Genet* **25**: 235–238.

Rotchell JM, Ulnal E, Van Beneden RJ, Ostrander GK. 2001. Retinoblastoma gene mutations in chemically induced liver tumour samples of Japanese medaka (*Oryzias latipes*). *Mar Biotechnol* **3**: S44–S49.

Rothenberg EV. 2001. Mapping of complex regulatory elements by pufferfish/zebrafish transgenesis. *Proc Natl Acad Sci USA* **98**: 6540–6542.

Sakamoto T, Danzmann RG, Gharbi K, *et al*. 2000. A microsatellite linkage map of rainbow trout *Oncorhynchus mykiss* characterized by large sex-specific differences in recombination rates. *Genetics* **155**: 1331–1345.

Setlow RB, Woodhead AD. 2001. Three unique experimental fish stories: *Poecilia* (the past), *Xiphophorus* (the present) and medaka (the future). *Mar Biotechnol* **3**: S17–S25.

Shima A, Shimada A. 2001. The medaka as a model for studying germ-cell mutagenesis and genomic instability. *Mar Biotechnol* **3**: S162–S167.

Sola L, Bressanello S, Rossi AR, *et al*. 1993. A karyotype analysis of the genus *Dicentrarchus* by different staining techniques. *J Fish Biol* **43**: 329–337.

Solnica-Krezel L, Schier AF, Driever W. 1994. Efficient recovery of ENU-induced mutations from the zebrafish germline. *Genetics* **136**: 1401–1420.

Taylor JS, Van de Peer Y, Braasch I, Meyer A. 2001a. Comparative genomics provides evidence for an ancient genome duplication event in fish. *Phil Trans R Soc Lond B* **356**: 1661–1679.

Taylor JS, Van de Peer Y, Meyer A. 2001b. Genome duplication, divergent resolution and speciation. *Trends Genet* **17**: 299–301.

Thorgaard GH, Bailey GS, Williams D, *et al*. 2002. Status and opportunities for genomics research with rainbow trout. *Comp Biochem Biophysiol B* (in press).

Tiersch TR, Chandler RW, Wachtel SS, Elias S. 1989. Reference standards for flow cytometry and application in comparative studies of nuclear DNA content. *Cytometry* **10**: 706–710.

Todorov JR, Elskus AA, Schlenk D, *et al*. 2002. Estrogenic responses of larval sunshine bass (*Morone saxatilis* × *M. chrysops*) exposed to New York city sewage effluent. *Mar Environ Res* **54**: 691–695.

Wester PW, Van der Ven LTM, Vethaak AD, Grinwis GCM, Vos JG. 2002. Aquatic toxicology: opportunities for enhancement through histopathology. *Environ Toxicol Pharmacol* **11**: 289–295.

Wisneski LA. 1990. Salmon calcitonin in the acute management of hypercalcaemia. *Calcif Tissue Int* **46**: S26–S30.

Wittbrodt J, Meyer A, Schartl M. 1998. More genes in fish. *BioEssays* **20**: 511–515.

Woods IG, Kelly PD, Chu F, *et al*. 2000. A comparative map of the zebrafish genome. *Genome Res* **10**: 1903–1914.

Wright JE Jr, Johnson K, Hollister A, May B. 1983. Meiotic models to explain classical linkage, pseudolinkage and chromosome pairing in tetraploid derivative salmonid genomes. *Isozymes Curr Top Biol Med Res* **10**: 239–260.

Young WP, Wheeler PA, Coryell VH, Keim P, Thorgaard GH. 1998. A detailed linkage map of rainbow trout produced using doubled haploids. *Genetics* **148**: 839–850.

Copyright © 2003 John Wiley & Sons, Ltd. 
